# Pylorectomy

**Published:** 1899-04-08

**Authors:** 


### PYLORECTOMY.

If we wish to understand the present position of surgery of the stomach, we may with advantage turn from the above to a series of cases exhibited at the last meeting of the Clinical Society by Mr. Rutherford Morison, illustrating the results of operation for pyloric obstruction. The first was a case of recovery after pyloroplasty for stricture of the pylorus. At the time of the operation in 1894 the patient had arrived at the condition of having to be fed entirely by the rectum. She is now in perfect health, and could eat anything. Mr. Morison mentioned that he had now performed this operation 19 times, and all the patients had recovered. Then came a series of five cases in which pylorectomy had been performed for malignant disease. These cases were very interesting, both from the size of the growths and the amount of tissue which in some of them had been removed. In one case there had been a scirrhous mass the size of an orange which could be felt near the umbilicus. The operation was performed in October, 1897. She could now eat anything, was in good health, and weighed one and a half stones more than before the operation. In another case there was an extensive colloid carcinoma involving the pyloric half of the stomach which was removed, and the cardiac end was joined to the duodenum by a modification of Billroth's method. A chain of glands was affected, and had to be removed. The patient was now in excellent health, and there were no signs of recurrence. One of the interesting points about this remarkable exhibition was that the cases were not selected, but consecutive ones. Everything seems to teach the same lesson, and from every side the evidence seems to be accumulating that so soon as a new growth is perceived it is safer to cut than to wait.

